# Successful Laparoscopic-Assisted Pancreaticoduodenectomy for a Neuroendocrine Tumor of the Papilla of Vater in Type 1 Portal Annular Pancreas

**DOI:** 10.70352/scrj.cr.25-0085

**Published:** 2025-06-03

**Authors:** Hideki Izumi, Hisamichi Yoshii, Rika Fujino, Kou Mikkaichi, Masaya Mukai, Junichi Kaneko, Hiroyasu Makuuchi

**Affiliations:** Department of Gastrointestinal Surgery, Tokai University Hachioji Hospital, Tokyo, Japan

**Keywords:** portal annular pancreas, laparoscopic-assisted pancreaticoduodenectomy, laparoscopic surgical procedures, pancreaticojejunostomy, postoperative pancreatic fistula

## Abstract

**INTRODUCTION:**

Portal annular pancreas (PAP) is a rare anomaly of pancreatic embryology that is classified into three types according to the position of the main pancreatic duct. PAP type 1, in which the main pancreatic duct runs dorsal to the pancreas, is extremely rare. Herein, we describe a case of successful laparoscopic-assisted pancreaticoduodenectomy in a patient with type 1 PAP.

**CASE PRESENTATION:**

A 72-year-old Japanese woman with neck swelling was referred to our hospital. CT at admission showed dilation of the main pancreatic duct. After a thorough examination, a preoperative diagnosis of carcinoma of the papilla of Vater was made. Neck swelling was attributed to a lymphoma for which chemotherapy was administered. Upon remission, CT imaging indicated PAP type 1, and a laparoscopic-assisted pancreaticoduodenectomy was performed. The retroportal pancreas was dissected just below the portal vein, but anastomosis was difficult; therefore, the pancreas was moved to the anterior surface of the portal vein, and anastomosis was performed. Postoperative pancreatic leakage occurred but was relieved by drainage, and the patient was discharged 26 days postoperatively. The postoperative diagnosis was neuroendocrine tumor of the papilla of Vater.

**CONCLUSIONS:**

Only one case of open pancreaticoduodenectomy for PAP type 1 has been reported previously. We successfully removed a neuroendocrine tumor from the papilla of Vater in a patient with PAP type 1 through laparoscopic-assisted pancreaticoduodenectomy and detailed the operative procedures for optimal outcomes in future cases.

## Abbreviations

AP
: anteportal pancreas
DP
: distal pancreatectomy
MRCP
: magnetic resonance cholangiopancreatography
PAP
: portal annular pancreas
PD
: pancreaticoduodenectomy
PV
: portal vein
RP
: retroportal pancreas
SMV
: superior mesenteric vein
SpV
: splenic vein

## INTRODUCTION

PAP is a rare anomaly of pancreatic embryology that occurs in 1.1%–3.4% of cases.^1,2)^ This anomaly is not a major problem when DP is performed for pancreatic body or tail tumor. However, when PD is performed, the site of pancreatic dissection and reconstruction method must be carefully considered.^3)^

PAP arises when the ventral pancreatic primordium rotates behind the anterior intestine during development and fuses with the pancreatic body to wrap around the left side of the PV.^1,4,5)^ Sugiura et al.^4)^ first identified PAP intraoperatively in 1987. Joseph et al.^5)^ classified PAP into three types based on the positional relationship between the main and accessory pancreatic ducts as follows: (I) the main pancreatic duct runs dorsal to the PV, (II) the main and accessory pancreatic ducts run dorsal and ventral to the PV, respectively, and (III) the main pancreatic duct passes ventral to the PV. Type 3, in which the main pancreatic duct runs ventral to the PV, is more frequent, comprising 63%–97.5% of cases,^6,7)^ but there are few reports of PD for type 3.^8)^ Type 1, in which the main pancreatic duct runs dorsal to the PV, occurs at a frequency of 8%–22%, while type 2, in which the main and accessory pancreatic ducts run dorsal and ventral to the PV, is least frequent at 0%–14.8%.^6,7)^

Recently, a minimally invasive type 3 PD was reported.^9)^ However, there is only one report of PD for PAP type 1 performed using an open laparotomy approach.^10)^ To our knowledge, this study is the first to report laparoscopic-assisted PD for PAP type 1 neuroendocrine tumor.

## CASE PRESENTATION

A 72-year-old Japanese woman was referred to the otolaryngology clinic with a chief complaint of neck swelling. She was diagnosed with Mantle cell lymphoma by biopsy. CT scan at that time showed dilation of the main pancreatic duct; therefore, a more detailed examination was performed, and a diagnosis of carcinoma of the papilla of Vater was made. Stage 4A Mantle cell lymphoma was treated first, with remission achieved after six rounds of bendamustine, a rituximab therapy. Thereafter, surgery was planned for carcinoma of the papilla of Vater.

**Fig. 1:**
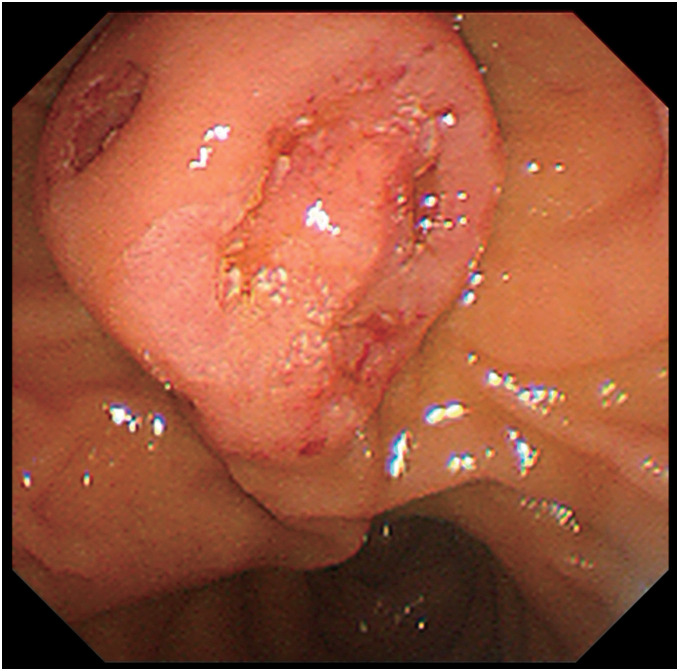
Endoscopic retrograde cholangiopancreatography image. Swelling is observed in the papilla of Vater, with ulceration observed below the orifice.

**Fig. 2:**
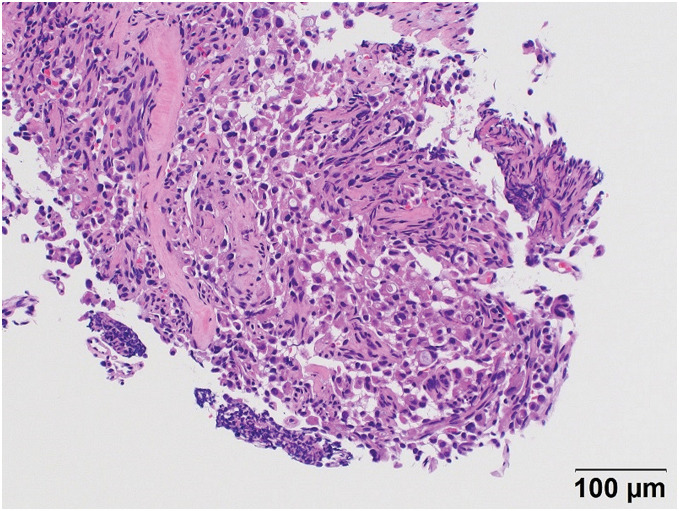
Hematoxylin and eosin staining. Carcinoma cells with eccentric nuclei infiltrating the mucosa in the duodenal biopsy specimen.

The patient had a history of atrial fibrillation, heart failure, hypertension, and appendicitis. No abnormal blood biochemical findings were noted, including tumor markers. Endoscopic findings showed swelling in the papillary region of the papilla of Vater with partial formation of an ulcer (**Fig. 1**). Carcinoma cells with eccentric nuclei were observed infiltrating the mucosa in the duodenal biopsy specimen. Intracytoplasmic lumina were also noted in some areas, and the tumor was diagnosed as a poorly differentiated adenocarcinoma (**Fig. 2**).

**Fig. 3:**
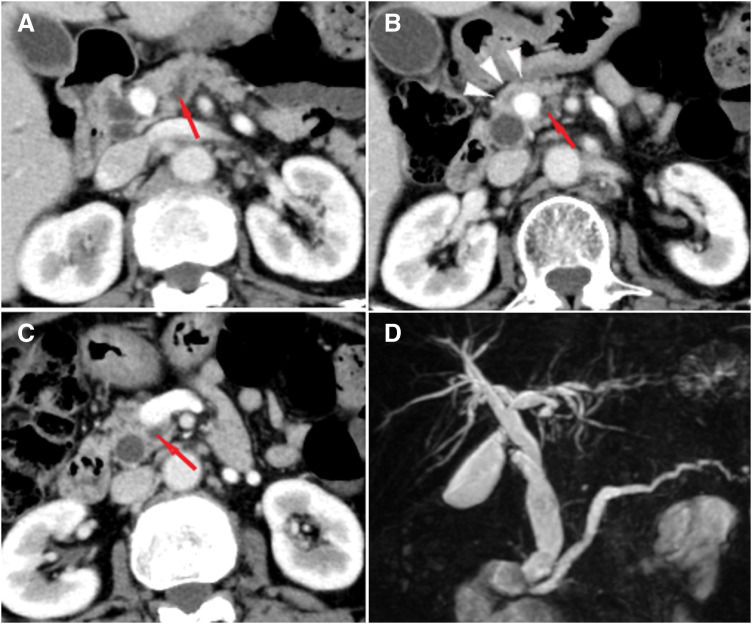
CT and MRCP images. (**A**) Dilation of the main pancreatic duct observed on CT imaging (red arrow). (**B**) The portal vein parenchyma is observed on the anterior surface of the pancreas (white triangle), but the dilated main pancreatic duct is located dorsal to the portal vein (red arrow). (**C**) Slightly dilated main pancreatic duct observed dorsal to the portal vein (red arrow). (**D**) MRCP shows dilatation of the main pancreatic duct with no obvious abnormality. MRCP, magnetic resonance cholangiopancreatography

Contrast-enhanced CT revealed dilatation of the main pancreatic duct (**Fig. 3A**–**3C**) located dorsally to the PV (**Fig. 3B**, **3C**). The pancreatic parenchyma was also present on the ventral side of the PV (**Fig. 3B**), leading to a diagnosis of PAP type 1. No distant metastases or lymph node metastases were observed. Magnetic resonance cholangiopancreatography revealed no abnormal findings and only a mild dilatation of the main pancreatic duct (**Fig. 3D**). Therefore, we performed laparoscopic-assisted PD with a diagnosis of carcinoma of the papilla of Vater (T2N0M0 stage IB) and PAP type 1.

### Operation results

**Fig. 4:**
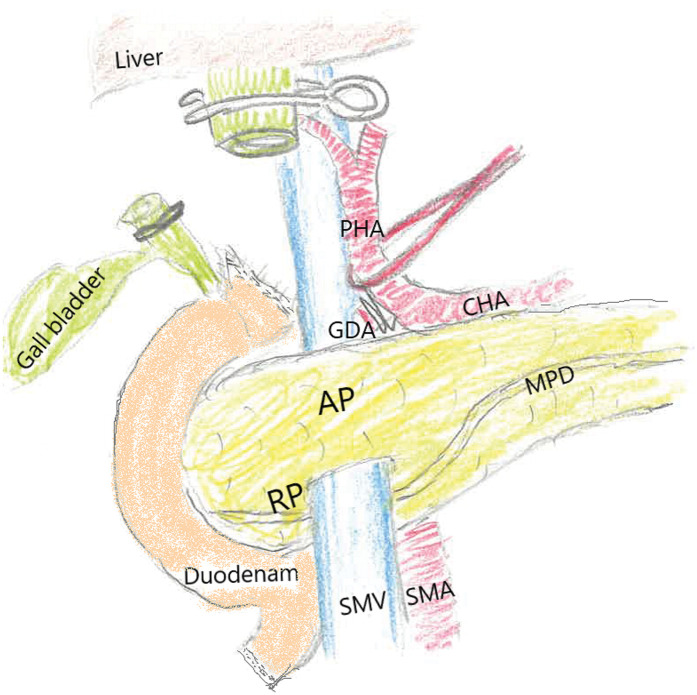
Anatomical relationship of the pancreas and portal vein, showing the main pancreatic duct coursing posterior to the portal vein.

Laparoscopic surgery began with five ports and comprised the following major steps: The gastrojejunal mesentery was opened, the superior mesenteric vein (SMV) was identified, and only the inferior part of the preportal pancreas was tunneled. A Kocher procedure was performed, and the duodenum, stomach, bile ducts, and gastroduodenal artery were dissected (**Fig. 4**). The last step was delineation of the pancreatic perimeter and dissection of the pancreas.

**Fig. 5:**
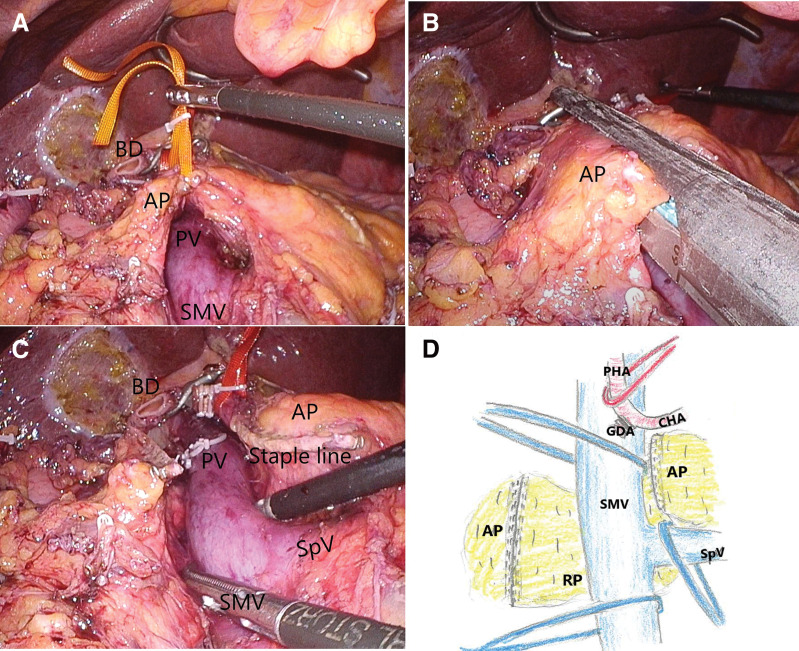
Laparoscopic images. (**A**) Tunneling and taping of the retroportal pancreas (RP) just above the PV. (**B**) Pancreatic dissection of the anteportal pancreas (AP) was performed using a Powered ECHELON FLEX. (**C**) Anteportal pancreas (AP) was dissected with a stapler. (**D**) The anteportal pancreas is dissected and taped to the vessels.

Tunneling of the anteportal pancreas was performed immediately at the beginning of surgery and subsequently taped (**Fig. 5A**). After confirming the anteportal pancreas by laparoscopic ultrasound and that the main pancreatic duct was not running, the anteportal pancreas was dissected just above the PV using an ECHELON + Stapling System (60-mm Green cartridge; Johnson & Johnson, Tokyo, Japan) (**Fig. 5B** and **5C**). Subsequently, the PV, SMV, and splenic vein were dissected and taped (**Fig. 5D**).

**Fig. 6:**
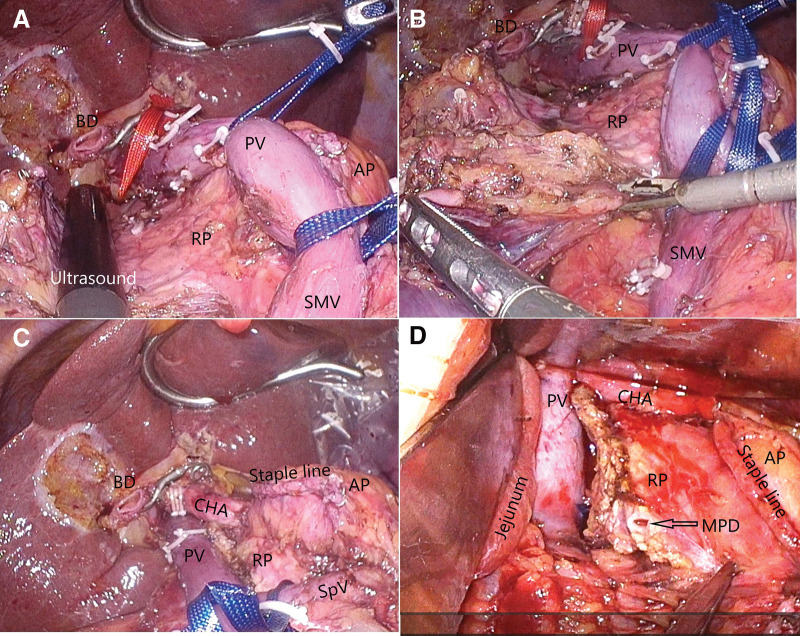
Laparoscopic images. (**A**) The PV and SMV are pulled to the left side of the patient, and an ultrasound of the retroportal pancreas (RP) is performed to confirm the location of the main pancreatic duct and bile duct. (**B**) The RP is dissected using an ultrasonic coagulation incision. (**C**) Following RP dissection, the surrounding area is dissected, and the RP is pulled out at the left margin of the PV. (**D**) Before anastomosis, a slightly dilated main pancreatic duct is observed on the dissected surface of the RP. PV, portal vein; SMV, superior mesenteric vein

The PV/SMV was moved to the left side of the patient, and ultrasonography was performed to confirm the location of the main pancreatic duct and bile duct of the retroportal pancreas (**Fig. 6A**). After determining the incision line, the retroportal pancreas was dissected using the Thunderbeat ultrasonic coagulation cutting device (Olympus Medical Systems, Tokyo, Japan) (**Fig. 6B**). The pancreatic parenchyma was dissected using the ultrasonic coagulation cutting device, while the main pancreatic duct was dissected using scissors. The main pancreatic duct was located dorsal to the PV, rendering the performance of the pancreaticoduodenal anastomosis difficult. Therefore, the retroportal pancreatic periphery was dissected such that the retroportal pancreas was placed in front of the PV (**Fig. 6C** and **6D**). A small hole of approximately 3 cm diameter was made in the mesentery of the transverse colon, and the jejunum was elevated for anastomosis.

A 5-cm incision was made in the upper abdomen, and the tumor was removed. All reconstructions were performed with direct visualization through the 5-cm incision using the modified Child reconstruction; bile duct choledochal anastomosis was performed first, followed by pancreatic duct jejunal anastomosis. As the main pancreatic duct was slightly dilated, pancreatic duct jejunal mucosa anastomosis was performed without a stent, and reconstruction was performed using the modified Blumgart method. Thereafter, gastric jejunal anastomosis was performed, and a closed negative-pressure drain was inserted dorsal to the jejunal anastomosis of the pancreatic duct. The operation was completed in 337 min with minimal blood loss (43 mL).

**Fig. 7:**
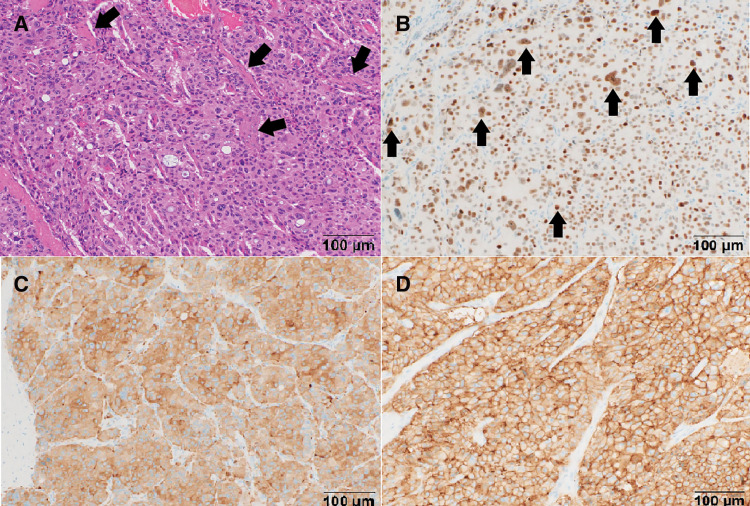
Hematoxylin and eosin staining and immunostaining. (**A**) The tumor cells with eosinophilic cytoplasm were proliferated in an alveolar pattern with rich vascularized stroma (black arrows). (**B**) Immunohistochemically, the tumor cells were positive for INSM-1 (black arrows). (**C**) The tumor cells were diffusely positive for synaptophysin. (**D**) The tumor cells were diffusely positive for the somatostatin receptor.

A nodular tumor occupied the duodenal papilla and infiltrated the smooth muscle fibers of the sphincter of Oddi. The tumor cells, characterized by eosinophilic cytoplasm, were proliferated in an alveolar pattern with a richly vascularized stroma. Intracytoplasmic lumina were occasionally observed. The nuclei showed size variation and atypia (**Fig. 7A**). Immunohistochemically, the tumor cells were positive for INSM-1 (**Fig. 7B**), synaptophysin (**Fig. 7C**), and somatostatin receptor (**Fig. 7D**), but negative for chromogranin A and CA19-9. Ki-67 labeling index was 2% at the hotspot. Based on these findings, the tumor was diagnosed as a neuroendocrine tumor, G2.

The postoperative course included the occurrence of a Grade B pancreatic fistula, as classified by the International Study Group of Pancreas Fistula. The fistula improved with drainage, and the patient was discharged 26 days postoperatively.

## DISCUSSION

PAP is usually asymptomatic but becomes problematic in pancreatectomy procedures, as the altered morphology increases the risk of pancreatic fistula.^11,12)^ Ohtsuka et al.^13)^ reported that 9 of 508 PD cases had PAP and that postoperative pancreatic fistula was more frequently observed in cases with PAP than in those without (44% vs. 14%, *p* = 0.03). Kiuchi et al.^14)^ retrospectively reviewed PD cases and reported that 7 of 552 (1.3%) cases had a PAP, which was associated with a significantly greater frequency of postoperative pancreatic fistulas (71%). Furthermore, in multivariate analysis, PAP was an independent factor for the occurrence of postoperative pancreatic fistula.^14)^ In typical PD procedures, a pancreatic incision is performed just above the PV or at the left margin if a sufficient resection margin can be secured from the tumor. However, in PAP, the pancreatic parenchyma dorsal to the PV must also be dissected at the same site, resulting in two dissecting interfaces. If the main pancreatic duct runs ventral to the PV in type 3 PAP, the dorsal fused pancreas can be ligated or closed with a stapler, and reconstruction can be performed in front of the PV. However, in type 1 and 2 cases, in which the main pancreatic duct runs dorsal to the PV, pancreatectomy near the PV requires reconstruction on the dorsal side of the PV, which is expected to cause difficulties in the reconstruction of the pancreatic duct. In these cases, the pancreatectomy site should be set caudal to the PAP fusion site to reduce the risk of a pancreatic fistula, and the pancreatectomy plane should be simplified to one.^13,15)^ In type 2 cases, in which the main pancreatic duct runs both dorsal and ventral to the PV, there are two pancreatic duct anastomoses. Therefore, the main pancreatic duct should be dissected caudally to unify the structure.^3)^ However, in type 1 cases, the pancreas on the anteportal side of the PV can be stapled off, and the main pancreatic duct on the retroportal side of the PV can be anastomosed.

This is the first known report of laparoscopic-assisted PD for type I PAP. Kawamoto et al.^10)^ reported a case of open PD in a patient with type I PAP secondary to pancreatic head cancer. They performed PD by disconnecting the anteportal pancreas just above the PV and pulling out the retroportal main pancreatic duct anteportal to the PV to preserve the remaining pancreatic function and avoid secondary damage. In the current case of a neuroendocrine tumor of the papilla of Vater, a similar technique was performed wherein the anteportal pancreas was dissected using a linear stapler immediately above the PV, and the retroportal pancreas was dissected immediately below the PV. Preoperatively, the retroportal pancreas and the main pancreatic duct were anastomosed directly to the dorsal PV. However, it was difficult to perform an anastomosis under a small incision because the PV was obstructed. Therefore, the anteportal area was dissected, and the retroportal pancreas was drawn ventral to the PV, allowing anastomosis to be performed with the usual field of view. PD for PAP is often reported to cause pancreatic leakage. In our case, pancreatic leakage also occurred, but it was treated by drainage.

Postoperative diabetes mellitus is a significant complication following pancreatectomy, adversely affecting patients’ quality of life. The incidence of postoperative glucose intolerance differs between PD and DP, with reported rates ranging from 21% to 26% for PD and 32% to 50% for DP.^16–18)^ Since insulin-secreting β cells are predominantly located in the caudal portion of the pancreas,^19,20)^ this likely explains the higher incidence of postoperative diabetes mellitus following DP compared with PD. Although there are no specific reports examining the incidence of diabetes mellitus in relation to the extent of pancreatic dissection in PD, it is reasonable to expect that the risk increases as the dissection line approaches the caudal region. Therefore, we dissected the pancreas just above and just below the PV in order to reduce the incidence of postoperative diabetes mellitus. Although we considered performing a caudal pancreatectomy to reduce the incidence of pancreatic leakage, as mentioned previously, the resection site should be set caudal to the PAP fusion site to reduce the risk of a pancreatic fistula, and the pancreatectomy plane should be simplified to a single transection.^13,15)^ However, we chose to preserve pancreatic function. Consequently, a pancreatic leakage occurred; however, it was successfully managed with drainage. Preservation of residual pancreatic function is very important in terms of postoperative residual pancreatic function.

## CONCLUSIONS

Only one case of open PD for PAP type 1 has been reported previously. We successfully removed a neuroendocrine tumor from the papilla of Vater in a patient with PAP type 1 through laparoscopic-assisted PD. This is the first known report of laparoscopic-assisted PD for type I PAP.

## ACKNOWLEDGMENTS

The authors would like to thank Tomoko Sugiyama M.D., Ph.D. and Takuma Tajiri M.D., Ph.D. for the pathological diagnosis. The authors also would like to thank Editage for English language review.

## DECLARATIONS

### Funding

None.

### Authors’ contributions

HI, HY, RF, KM, MM, JK, and HM performed surgery and postoperative management.

All authors have read and approved the final version of this manuscript.

### Availability of data and materials

Not applicable.

### Ethics approval and consent to participate

This work does not require ethical considerations or approval. Informed consent to be included in this study was obtained from the patient. This study and procedures were carried out in accordance with the international clinical guidelines and the Declaration of Helsinki.

### Consent for publication

The patient provided consent for publication of the case report and all accompanying images.

### Competing interests

The authors declare that they have no competing interests.
